# Distribution and utilization of homestead windbreak Fukugi (*Garcinia subelliptica* Merr.) trees: an ethnobotanical approach

**DOI:** 10.1186/s13002-021-00434-3

**Published:** 2021-02-22

**Authors:** Bixia Chen, Hikaru Akamine

**Affiliations:** 1 Department of Subtropical Agriculture, Faculty of Agriculture, University of the Ryukyus, 1 Senbaru, Nishihara Town, Okinawa, 903-0213 Japan; 2 The United Graduate School of Agricultural Sciences, Kagoshima University, 1-21-24, Korimoto, Kagoshima, 890-0065 Japan

**Keywords:** Cultural keystone species, Ethnobotany, Heritage trees, Ornamental plant, Windbreak

## Abstract

**Background:**

*Garcinia subelliptica* (Fukugi in Japanese) is an evergreen tropical tree, first identified in Batanes, the Philippines, which has been planted as a homestead windbreak and in coastal forests extensively on the Ryukyu Archipelago, Japan. This article focuses on the traditional uses and cultural values of Fukugi trees and provides ethnobotanical information for the conservation scheme of this important tree species.

**Methods:**

A combination of ecological and ethnobotanical approaches was applied in this study. Extensive field surveys were conducted to collect dimensions of relatively large trees, and in-depth interviews with the village leaders and knowledgeable persons were conducted to collect ethnobotanical data.

**Results:**

Fukugi trees have been primarily planted as homestead or farmland windbreaks. Timber was harvested during difficult times, for example, after WWII, and used for recreational purposes for children or farmers. The fruits were also eaten on some remote islands. Old-growth Fukugi trees are widely found in sacred sites, within cities, and as symbolic trees. The older generations respect Fukugi trees; however, the cultural significance valued by older generation seems to be lacking in the younger generation. We argue that Fukugi is a cultural keystone species in Okinawa, which underpins Ryukyu culture and has transformed islands into a pleasant land, a unique place, and shared identity for the community.

**Conclusions:**

Fukugi windbreaks provide significant ecosystem services, such as biodiversity in the forest, reducing soil erosion, and spiritual and cultural values. A combination of biophysical environment, as well as tradition and custom, has played an essential role in tree species selection for windbreaks. The positive impacts that anthropogenic activities have had on the sustainability of woody species, namely, the active utilization of tree species, may have enabled the species to sustain. Strategies for protecting old-growth Fukugi trees, in addition to restoration of damaged trees, are needed to improve the sustainable management of Fukugi trees in Okinawa.

## Introduction

*Garcinia subelliptica* Merr***.*** (Fukugi in Japanese) trees have been planted as homestead windbreaks and in coastal forests over a wide range in the Ryukyu Archipelago, Okinawa Prefecture, as well as in the southern part of Kagoshima Prefecture, Japan [1]. Homestead windbreaks are managed as part of a residence and are strips of trees planted and maintained to alter wind flow and microclimate. In addition to Fukugi trees, other plant species were also used as windbreaks, among which *Diospyros egbert-walkeri* was the most common, while *Podocarpus macrophyllus* and *Calophyllum inophyllum* were also planted [2]. It is believed that such rural landscapes with planted trees, especially Fukugi trees as windbreaks, were designed based on Feng Shui concepts in the Ryukyu Kingdom, around 300 years ago [3–5].

Fukugi trees belong to the genus Garcinia of the Clusiaceae family, which is native to the tropics and subtropics in Asia and Africa. Plants of the genus are ornamental, with a dense canopy of green leaves. Fukugi trees are evergreen tropical trees that are 10–15 m tall when mature. Fukugi trees are highly distinctive because they have only one main trunk that supports alternating pairs of erect branches, producing compact and conical crowns. These trees are planted as windbreaks in Okinawa due to their compact upright form [6]. Leaves are spirally arranged in opposite pairs, and are thick, glossy, and ovate-oblong, and fruits are depressed-globose and orange [6].

Fukugi can be literally translated as “happiness tree”; however, it is unclear why it is called Fukugi in Okinawa. Fukugi is pronounced differently in local languages, except in some regions [7]. For example, it is called *saba gi* in North Okinawa Island, which means “sandal tree”; Fukugi tree has opposite pairs of leaves, which could be worn as sandals by children. Additionally, it is called *kuwajii gi*, meaning a tree that can prevent fires.

There are over 300 species belonging to the genus Garcinia, which have received attention in the pharmaceutical industry for immense remedial qualities and have been used in ayurvedic preparations in medicines for various pathophysiological disorders [8]. Several active chemical compounds have been discovered in the fruit, seed, leaf, wood, bark, and roots of Fukugi trees. The major compounds contained within *G. subelliptica* are benzophenone, xanthones, biflavonoids, and triterpenoids [9]. Fukuyama et al. [10] isolated Garsubellin A from the wood of *G*. *subelliptica*. Garsubellin A has potential in developing therapies for Alzheimer’s disease and exhibits anti-inflammatory properties through its inhibition. However, no medical applications have yet been developed from Fukugi trees.

In line with global environmental problems, homestead windbreaks on the islands are rapidly deteriorating due to modernization, rapid urbanization, and other anthropogenic factors, as well as natural disasters [11]. Hence, an appropriate strategy is needed to conserve and sustainably utilize this highly valued tree species. Additional baseline information on plant species and ecosystems can support sound scientific approaches for conservation schemes [12].

Numerous publications have demonstrated the importance of ethnobotany in the conservation and management of trees and forests (e.g., [13, 14]). Ethnobotanical studies of a specific plant species can provide a holistic lens for the relationships between people and plants by involving the integration of many disciplines including anthropology, botany, and ecology, among others [15, 16]. This will enable a better understanding of the traditional knowledge and use of a plant species by improving management and conservation policies [17–21]. Previous studies have revealed the spatial layout of Fukugi windbreaks inside a village [3–5] and conservation issues related to this tree species [11, 22]; however, its cultural and spiritual values have not yet been systematically studied. In this article, we focused on describing the traditional uses and cultural values of Fukugi trees and to provide ethnobotanical information for the conservation of this important tree species. In specific, we aimed to (1) document the traditional ethnobotanical uses of Fukugi trees, and their cultural and spiritual values, as perceived by local people; (2) clarify the ecological distribution of Fukugi trees in the Ryukyu Archipelago and outside of Japan; and (3) identify critical actions for the sustainable management of Fukugi trees. This will provide ethnobotanical information for conservation schemes for this important tree species and will encourage the preservation of the cultural, traditional, and sustainable utilization of this tree species. Moreover, it will provide useful knowledge related to the valuation of other small-scale forests and other tree species that have been planted in the vicinity of human settlements in the world.

## Methods

### Survey sites

**Fig. 1:**
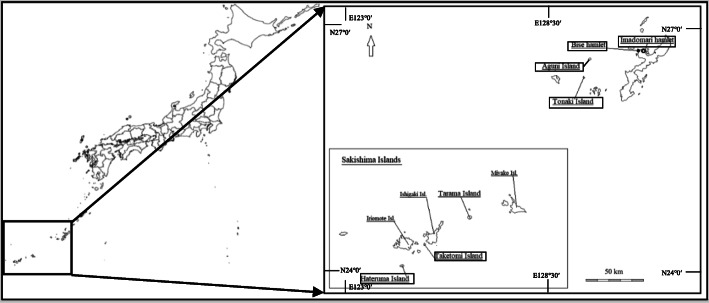
Location of the Ryukyu Archipelago

Okinawa Prefecture, which is located in the southernmost part of Japan, encompasses two thirds of the Ryukyu Archipelago, and extends over 1000 km (Fig. 1). It consists of 39 inhabited islands and 121 uninhabited islands. Okinawa Prefecture has an area of 2280.9 km^2^. The population was 14.5 million in July 2019.

The Okinawa climate is influenced by the latitude, surrounding ocean, the Black Current, monsoons, and typhoons. Okinawa belongs to the subtropics and has very mild winters with long, muggy, and rainy summers. From June to October, Okinawa can be affected by typhoons. The long-term average annual temperature of the Okinawa area has been rising at a rate of 1.16 °C per 100 years [23]. Extreme high temperatures have increased, while extreme low temperatures have decreased in the past 100 years [23].

The previous Ryukyu Kingdom ruled the islands of the current Okinawa Prefecture and south of the Kagoshima Prefecture. Hence, the traditions and cultures in Okinawa Prefecture are distinct from those in mainland Japan. Three island groups in the Ryukyu Archipelago from north to south are the Amami Islands, Okinawa Islands, and Sakishima Islands. The sites surveyed in the present study were distributed across the archipelago.

### Survey methods

A combination of ecological and ethnobotanical approaches was used in this study. Field surveys as well as secondary data, including official reports and published papers, were used as data sources.

### Tree survey and tree age estimation formula

Quantitative data was measured in 2008–2010 and 2014–2017 on the Ryukyu Archipelago (Table 1). Methods consisted of measuring the diameters and heights of all trees. Cultural values of plants may be geographically specific and unique. In order to verify the Ryukyu cultural use of Fukugi trees, the distribution of old-growth Fukugi trees outside of Japan was surveyed in the Philippines in April–May 2016. The species is also native and naturally distributed in the tropical areas of many countries, for example, Indonesia and China, but was sparsely distributed. Tao people in Lanyu (Orchid) Island, Taiwan, used to harvest *Garcinia subelliptica* in the lowland forest to build the bottom keels of large fishing boats, and used thin branches for fishing net frames and the main stem as the roof of the boats [24]. Currently, only a small number of *G. subelliptica* can be found in native forests on Lanyu Island and Ludao Island [24]. Our purpose was to examine the cultural uses of the species. Hence, only Taiwan and Batanes Island were selected for our investigation for two reasons: (1) several flora books (e.g., [6, 25, 26]) mention that Fukugi originated in Batanes and was distributed in these regions, and (2) these two regions have similar climate and frequency of typhoons. The first author and her colleagues spent 4 days searching for Fukugi trees on Batanes Island, which is located north of the Philippines. A field surveyor was hired from the Batanes provincial government along with a college teacher as a research assistant. A field trip to Taiwan was undertaken in November 2016 by one researcher and one research assistant, accompanied by a local researcher from the Forest Research Institute.

**Table 1:** The number and dimensions of remnant Fukugi trees in survey sites

		Number of remnant Fukugi trees at each estimated year range								Estimated age of largest tree (years)	Mean tree height (cm)	Mean DBH (cm)	Survey date
		Total	≥ 300 years	250~299 years	200~249 years	150~199 years	100~149 years	50~99 years	0~49 years				
Motobu Town	Bise^a^	1075	1	17	89	360	609			300	994^*2^	38.5	Aug. 2008
Tonaki Village	Tonaki Isl.^a^	965	0	2	10	111	842			268	842^*2^	31.13	Aug. 2009
	Tonaki Isl.^b^	7708	0	2	10	111	842	4918	1825	268	617	17.28	Oct. 2009
Nakijin Village	Imadomari^a^	1293	0	15	85	307	886			294	900	35.18	Jun. 2009
Aguni Isl.	East and West^a^	2561	0	16	82	486	1,977			296	723	33.17	Mar. 2009
	Hama^a^	500	0	3	6	55	436			281	713	31.27	Apr. 2009
Miyako city	Tarama Island, Shiogawa hamlet^a^	1089	0	1	8	157	923			257	1010	32.1	Aug. 2010
Miyako city	Tarama Island, Nakashiji hamlet^a^	1529	0	1	17	240	1334			262	1030	32.2	Aug. 2010
Taketomi town	Taketomi Island^b^	1183	1	13	50	143	298	255	423	304	605	22.7	Nov. 2017
	Hateruma Isl.^b^	2825	0	3	52	463	1278	841	188	266	720	28.2	Aug. 2014, Aug. 2015

^a^Only Fukugi trees with DBH larger than 25 cm were measured

^b^Fukugi trees with DBH larger than 5 cm were measured

The age of Fukugi trees was estimated based on tree diameter at breast height (DBH) using Eq. (1), derived by Hirata [27], and Eq. (2) by Nakama et al. [28].

1 $$ y={x}_1\div 2\times 8 $$

2 $$ y={x}_2\div 2\times 6.2 $$

where *y* is the estimated tree age*, x*_1_ is the DBH (cm) at 1.3 m above ground level, and *x*_2_ is the diameter (cm), approximately 0.2–0.3 m above ground level. We adopted Hirata’s method (Eq. (1)) because the diameter measurement at 0.2–0.3 m above ground level was not available for many trees (because the lower parts of their stems were surrounded by and buried in stone fences). In addition, DBH is an important parameter in a vegetation survey for tree size measurement.

#### Qualitative survey and analysis methods

Ethnobotanical investigations were conducted as part of a long-term research project of Fukugi tree distribution in a wide area in Okinawa Prefecture and the southern part of Kagoshima Prefecture. These areas were under the political control of the then Ryukyu Kingdom and were influenced by Ryukyu culture.

Semi-structured interviews and participatory observations have been applied to collect qualitative data in 2015, 2016, and 2020. Community leaders and knowledgeable senior residents were interviewed. The questions included the local name, the traditional uses, and their life stories with the species. We conducted the interviews based on feasibility and a snowball sampling method, depending on the introduction by the village heads, staff in the village offices, and non-profit organizations (NPOs). At first, the first author contacted the village office or community leaders, and then, knowledgeable people were identified and approached for interviews. The first author contacted several villagers for the survey; however, many women were hesitant to express their opinions before a stranger. Finally, a total of 26 major interviews, listed in the Appendix, were conducted with respondents from six villages of five islands. Each major interview was transcribed verbatim, and we cited the interviewee’s words, while sometimes adding a few words in parentheses, to ensure the completion and readability of the interviews. A dozen informal interviews were also conducted to enrich ethnobiological qualitative information on land use and management issues.

Qualitative data collected from the responses were coded to calculate the importance of each use in the people’s life in the island. The traditional uses of Fukugi trees were coded into 12 categories. The number of times each informant mentioned a particular use was counted and added to calculate the frequency of citation (FC) and the relative frequency of citation (RFC), based on Eq. (3), according to [29].

3 $$ \mathrm{RFC}=\frac{\mathrm{FCs}}{N} $$

where RFC is the relative frequency of citation; FCs is frequency of citation, and *N* is the total number of informants.

## Results

### Ecological aspects of distribution

**Fig. 2:**
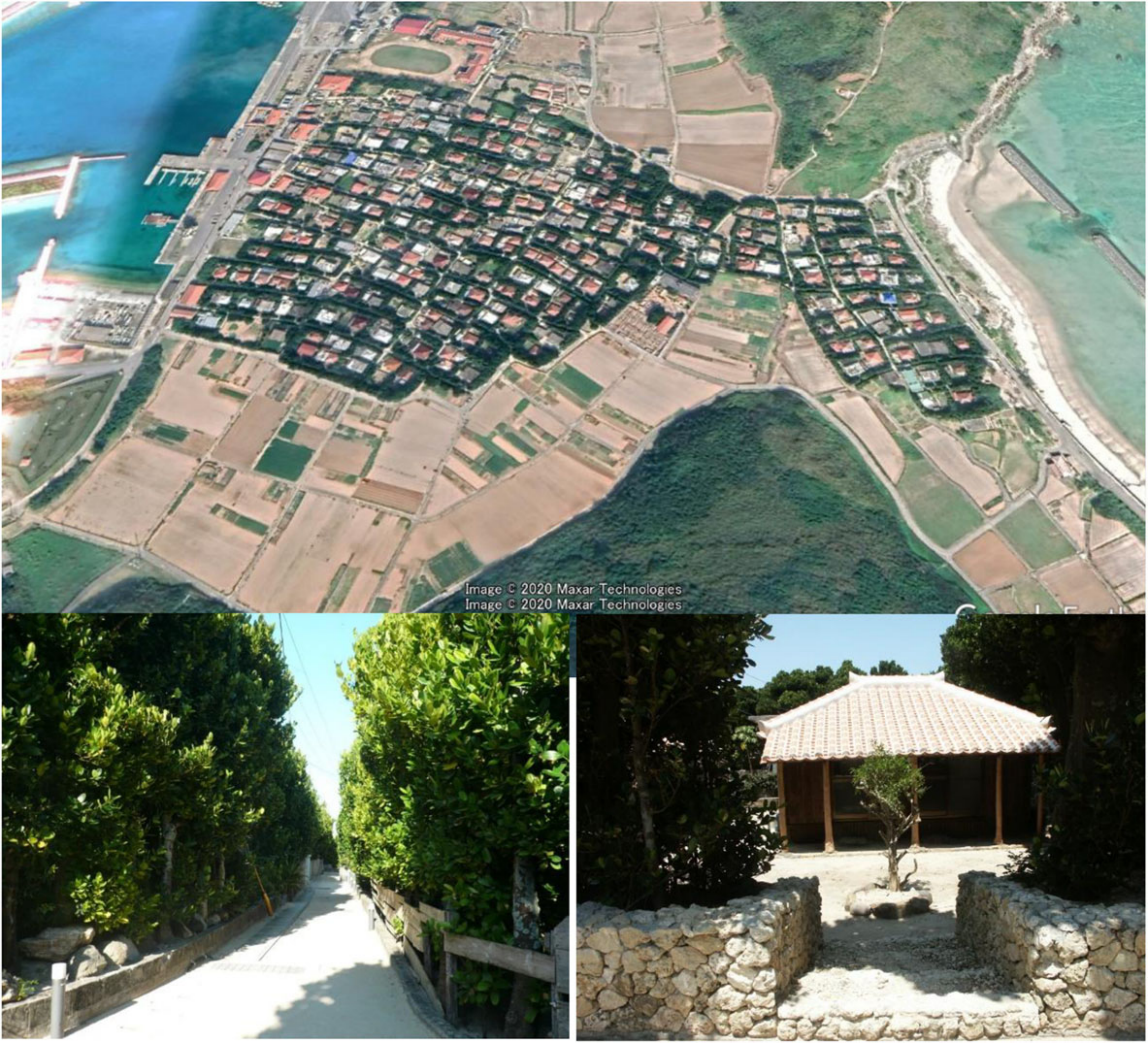
Above photo: a panoramic view of Tonaki Village landscape. Bottom (left): Windbreak trees line along the sandy road. Bottom (right): A red-tiled roof wood house embraced by Fukugi trees lines and coral stone fence outside

According to the Flora of Ryukyu [25], Fukugi trees are widely planted as windbreaks on the Ryukyu islands. Old-growth Fukugi trees exist prevalently around residences in traditional villages in Okinawa Prefecture, Japan. Fukugi tree dimensions in the survey sites are presented in Table 1. It is considered that such a rural landscape with planted Fukugi trees, and other trees as windbreaks, was designed based on the Fengshui concept in the then Ryukyu Kingdom, around 300 years ago. In some villages, around 70% of all the houses were surrounded by Fukugi trees. In Bise village, north of Okinawa Island, around 10,000 Fukugi trees were counted [22], among which 1075 Fukugi trees were estimated to be older than 100 years [5]. On Tonaki Island, an isolated small island near Okinawa Island, we counted a total of 7700 Fukugi trees, among which around 1000 trees were older than 100 years (Fig. 2). There are other several villages in mainland Okinawa and its nearby isolated islands with around 1000 Fukugi trees older than 100 years [5].

Our field surveys on the islands revealed that Fukugi trees primarily existed as homestead windbreaks on the Amami Islands, Okinoerabu, and further south on the islands in Okinawa Prefecture. On Okinoerabu’s more northern islands, Fukugi trees were sparsely distributed as windbreaks [5]. Banyan trees (*Ficus microcarpa*) were commonly found as windbreaks and landscaping trees [5].

In addition to the planted Fukugi trees, some naturally generated trees were also found on the hill slopes of remote islands, such as Iriomote Island [25], Yonaguni Island, and islands north of Okinawa Island. However, natural Fukugi trees are usually small in size and sparsely distributed in coastal areas or uphill slopes behind village settlements. Outside of Japan, old-growth Fukugi trees that were planted were only found in Taiwan based on our limited field survey until now. Fukugi trees planted in 1901 as landscaping trees were found in Kangting National Park, where the largest tree had a DBH of 61 cm and was 15 m tall. Further surveys of field trips to Taiwan and secondary data surveys of Fukugi uses in the Batanes Islands and other countries in Southeast Asia are required to clarify the historical and cultural importance of *G. subelliptica* in human life.

*G. subelliptica* originated in Batanes, the Philippines [26]; however, in these islands, we did not find Fukugi trees used as homestead windbreaks or close to human settlements or developed areas. The scientific name of Fukugi, *Garcinia subelliptica*, is named after Laurent Garcin, an eighteenth century French botanist, who first collected specimens of this species from Batanes, the Philippines. In 1908, Merrill [30] reported that one tree had a trunk 35 cm in diameter, with yellowish branchlets growing in thickets along the Philippine seashore. In Basco, the capital city of the province of Batanes, *Podocarpus macrophyllus* trees were planted in homestead windbreaks. We spent only 4 days in Batanes in June 2016; however, we could not find any Fukugi trees in the coastal areas on the island of Batanes. *G. subelliptica* may not be commonly used by people on Batanes Island. This may have resulted from the dramatic landscape transformation into grassland for cattle grazing, and into other areas that are not being actively used by the local people.

### Ethnobotanical uses

The traditional uses of Fukugi trees were coded into 12 categories, and the importance of Fukugi trees in people’s lives is summarized in Table 2. All respondents commented on the use of Fukugi tree as a windbreak. The respondents indicated that Fukugi trees provide diverse functions, such as amenity landscape, timber, ritual ceremony, and cooling air inside the homestead (Table 2).

**Table 2:** Traditional uses pertinent to their relative citation frequencies (RFC)

Coding	RFC (%)
Homestead windbreak	100
Homestead firebreak	19
Tree belts prevention from tidal water	4
Landscape amenity	27
Timber	19.2
Dyeing	15.4
Windbreak for the home garden	7.7
Cooling the air inside the homestead	11.5
Praying for the gods	7.6
House borderline	5
Eating fruits	4
Green manure for crops and as soil improvement material	11.5

In terms of respondents’ age, among the 26 respondents, we were able to access only two respondents in their 30s, one in their 40s, and two respondents were in their 50s, while the other 20 respondents were over 60 years old (see the Appendix). Further, the younger respondent, who was in his 30s, stated far fewer uses of Fukugi trees than the respondents who were older than 80.

Fukugi trees in Okinawa were primarily used for homestead and farmland windbreaks. Okinawa has a subtropical monsoonal climate, which suffers from frequent strong typhoons in the early summer to late autumn and winter wind from the north. Windbreaks are essential to crop planting and to people’s houses. In the past, traditional houses were made from timber and thatched roofs, which was the reason why homestead windbreaks were prevalent on the islands. Nowadays, the transformation of house building in concrete contributes to the cutting of many windbreaks in Okinawa. However, many people still cherish their traditional windbreaks.

One interviewee, K. (male, 68 years old, Bise hamlet), described the indispensable role of Fukugi trees as house windbreaks. He declared that “without Fukugi trees, we would not be able to live in Bise (hamlet). The Fukugi tree is green all year round. The north wind is very strong.” Fukugi windbreaks are also believed to be superior to concrete walls, although the Fukugi hedgerows have been removed and replaced by concrete walls on many islands. Another interviewee, M. (male, 50s, Aguni Island) told us in detail that in Aguni Island, Fukugi tree lines standing together with porous coral stone fence can absorb (mitigate/weaken) the damaging power of strong winds. He recalled the case of a neighborhood grandmother, who changed to using a concrete wall as windbreak and found that the winds became stronger before hitting the house.

Old-growth trees were important timber sources in remote islands, especially during the period immediately after WWII when resources and materials were depleted due to the war. S. (male, 60 years old, Hateruma Island) said that approximately 80% of the timber was from the neighboring Iriomote Island. Old windbreaks in most of the survey sites were selectively harvested and the trunks were submerged in sea water to develop insect repellent properties when used as flooring and poles. Old respondents (M.S., male, 90s; M.R., male, 90s) from Taketomi Island also stated that island people extracted *G. subelliptica* from the mountains of Iriomote Island, which was prohibited by the Forestry Agency after the Iriomote Islands was designated as a National Park in 1972. M.R. said that the big Fukugi windbreaks fell when his father rebuilt their former house. He also stressed that the trees standing at the four corners of the homestead must not be cut, because the spirits accommodating the old trees protect the house. If the trees in the corners were cut, the family would meet with misfortune.

In addition to being used as windbreaks, Fukugi trees contribute to agricultural production in two ways: as green manure for crops and as soil improvement materials used to fill in land. In remote small islands, there are very few natural forests that farmers can use for their farmland. Fukugi leaves can be buried in farm soil to decompose into humus or burned to improve soil fertility.

Interviewees also described Fukugi leaves as being used as toys and as trees providing shade for breaks during hot summers. M. (66, male, Bise hamlet) said that “In summer, it is cool under the trees as it provides shade from strong sunshine. We ate lunch under the trees and take a nap, then went to farm work at 4:00 pm.” Many senior residents from islands north of Okinawa Island vividly recalled memories of their childhood when children often used to cut one leaf from a pair of leaves to make leaf sandals to play with their friends around the village.

In the southern islands of Okinawa Prefecture, many informants over 60 years old stated that they ate the fruits during their childhood. Fruits ripen in August and are similar to oranges with four or five seeds. Very few fruits are presently eaten, and many residents do not know that the fruits are edible, after the import of fruits from overseas and mainland Japan in recent decades.

**Fig. 3:**
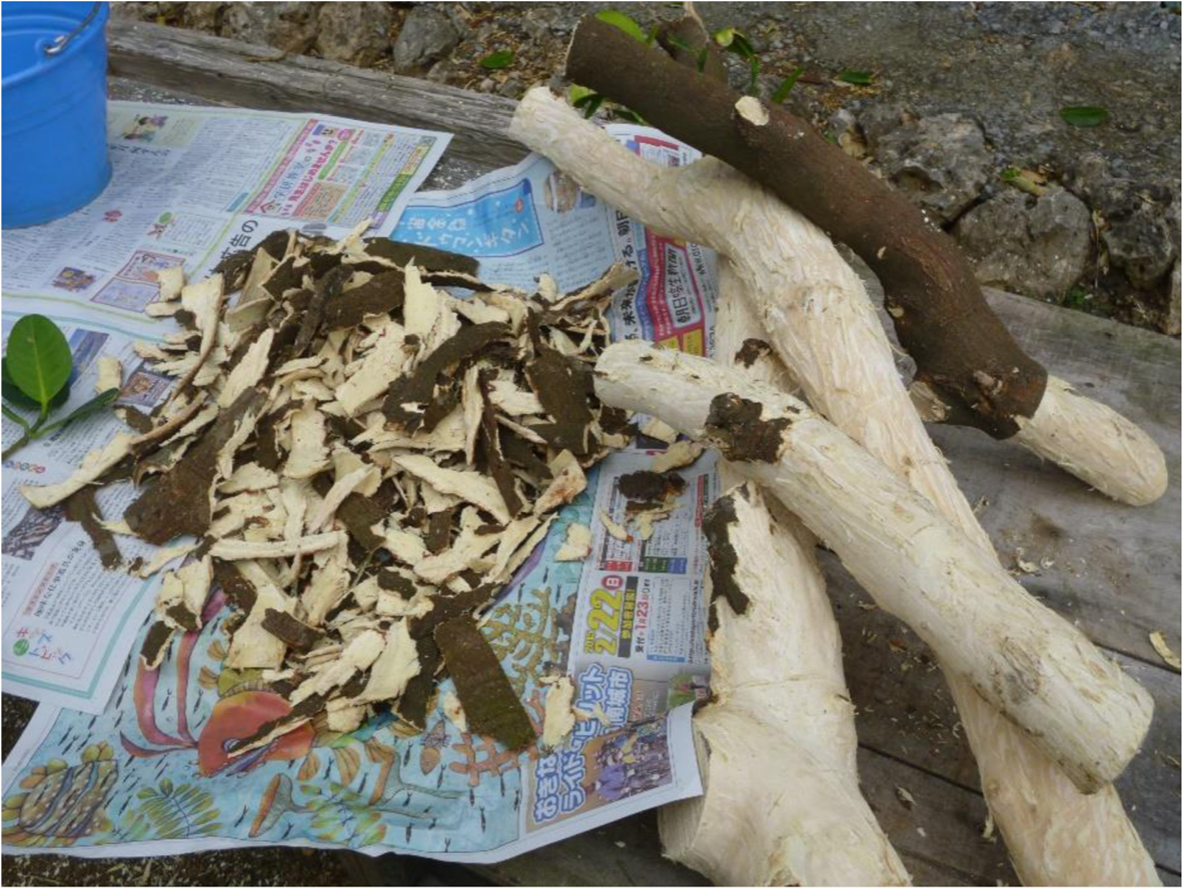
Bark from Fukugi tree stem dried as a dyeing material

**Fig. 4:**
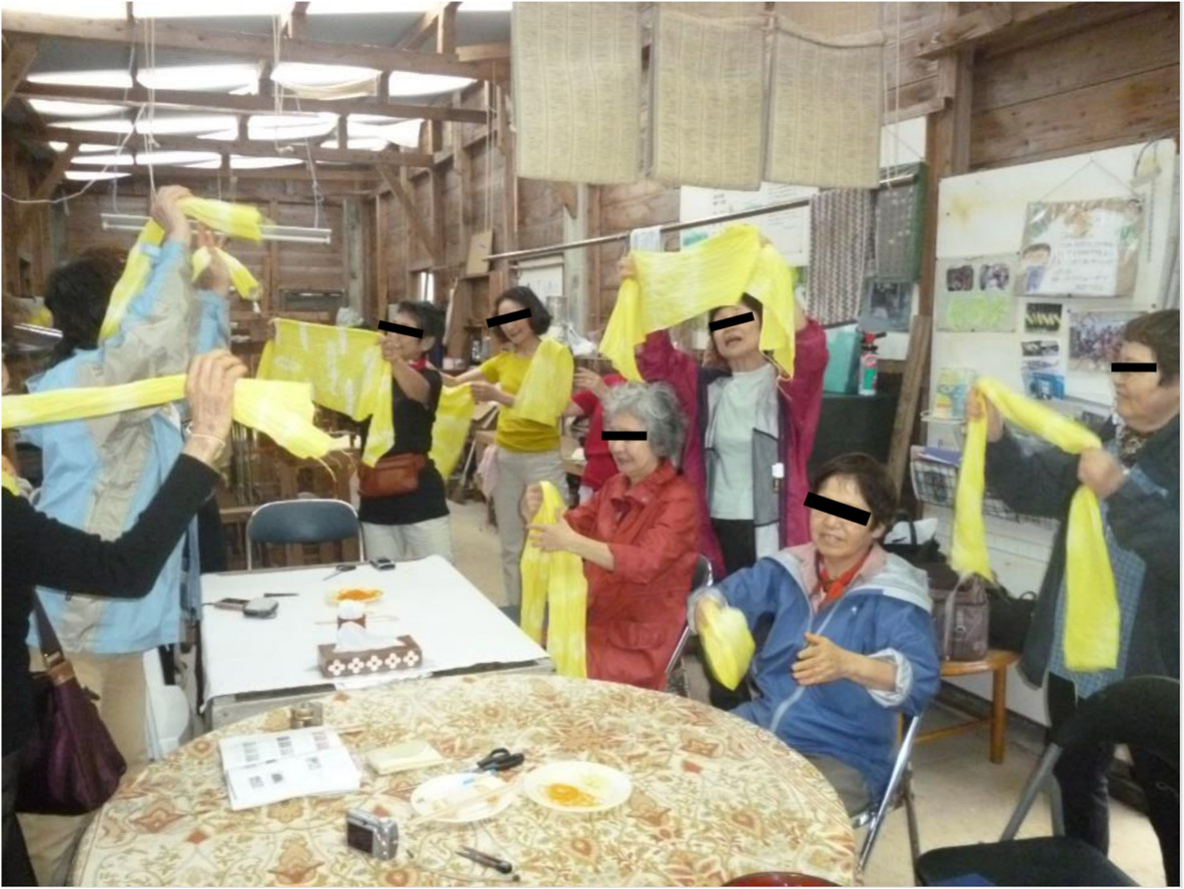
Fukugi dyeing class for the aged people group

Fukugi trees are important dye-producing plants in the Ryukyu Islands. Historically, Fukugi trees have been widely used to extract yellow dyes for textiles, especially for costume fabrics used in traditional festivals and everyday life. Recently, hands-on workshops have been held for groups of older people and local children (Figs. 3 and 4). Such workshops can potentially be held for tourists, since about 10 million tourists currently visit Okinawa each year.

### Sacred trees

Fukugi trees also contribute to a local community’s spiritual life as sacred forests, either as pure Fukugi forests or mixed in forests with other native tree species. For most sacred sites, one or a small number of big Fukugi trees still exist; however, groves of big Fukugi trees were found in the sacred sites *utaki* and *ashagi* (Table 3). In Okinawa, a hamlet is based on a territorial band, as well as a kinship and community. Each hamlet has its own sacred places of *utaki, ashagi*, or *haisho*. *Utaki* are usually located on the outskirts of hamlets and are places for the veneration of guardian gods and ancestors. *Ashagi* or *haisho* are always located inside hamlets, usually at the center, where guardian gods were summoned to hold ceremonies and rituals, with the harvest festival being the most common. Within approximately 37 hamlets, *utaki*s and/or other sacred sites had large remnant Fukugi trees. The oldest tree found in the sacred sites was in the *utaki* of the hamlet of Furugen, Yomitan Village, and is estimated to be 344 years old. The *ashagi* in Nakaoshi contained the oldest Fukugi trees. A large Fukugi tree that is 12 m tall and 360 years old is at the southern entrance to the Kannonji (the Goddess of Mercy) Temple, Kim Town, in the middle part of Okinawa Island. The other three sacred sites are located inside the hamlets surrounded by Fukugi trees.

**Table 3:** The biggest Fukugi trees found in the sacred sites of *utaki* and *ashagi*

*Utaki/Ashagi*	Tree number	DBH (cm)		Estimated tree age (years)		Tree height (cm)	
		Maximum	Average	Maximum	Average	Maximum	Average
Tōbaru	21	76.6	47.4	282	189	1172	897
Hiji	20	66.1	42	264	168	-	1480
Nakadomari	20	62.5	42.1	250	168	1550	1450
Furugen	12	86	49.5	344	198	1306	1048

Tōbaru utaki and ashagi were built in the same place inside the hamlet with surrounding Fukugi trees

Hiji *ashagi* is a thatched roofed small building covered by a thick forest, located on the top of the hill behind the hamlets. In addition to planted Fukugi trees, large *Bischofia javanica* trees were also present

Nakadomari ashagi is located in the middle of the village and is now used as a public playground and for annual sacred ceremonies

Furugen utaki is located inside the village, covered by a grove of a pure Fukugi forest

**Fig. 5:**
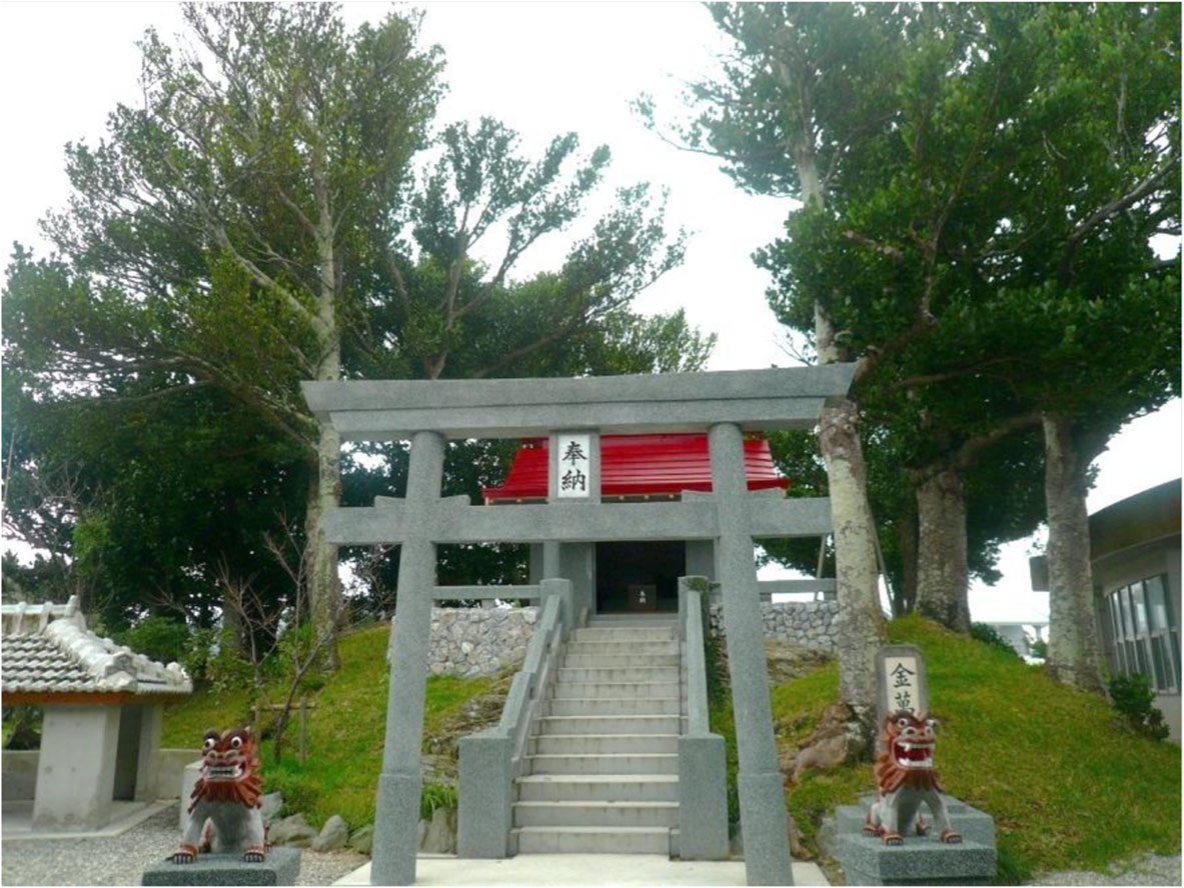
A sacred site of *utaki* inside Tōbaru hamlet, north of mainland Okinawa might be the site of an earliest house in the hamlet

**Fig. 6:**
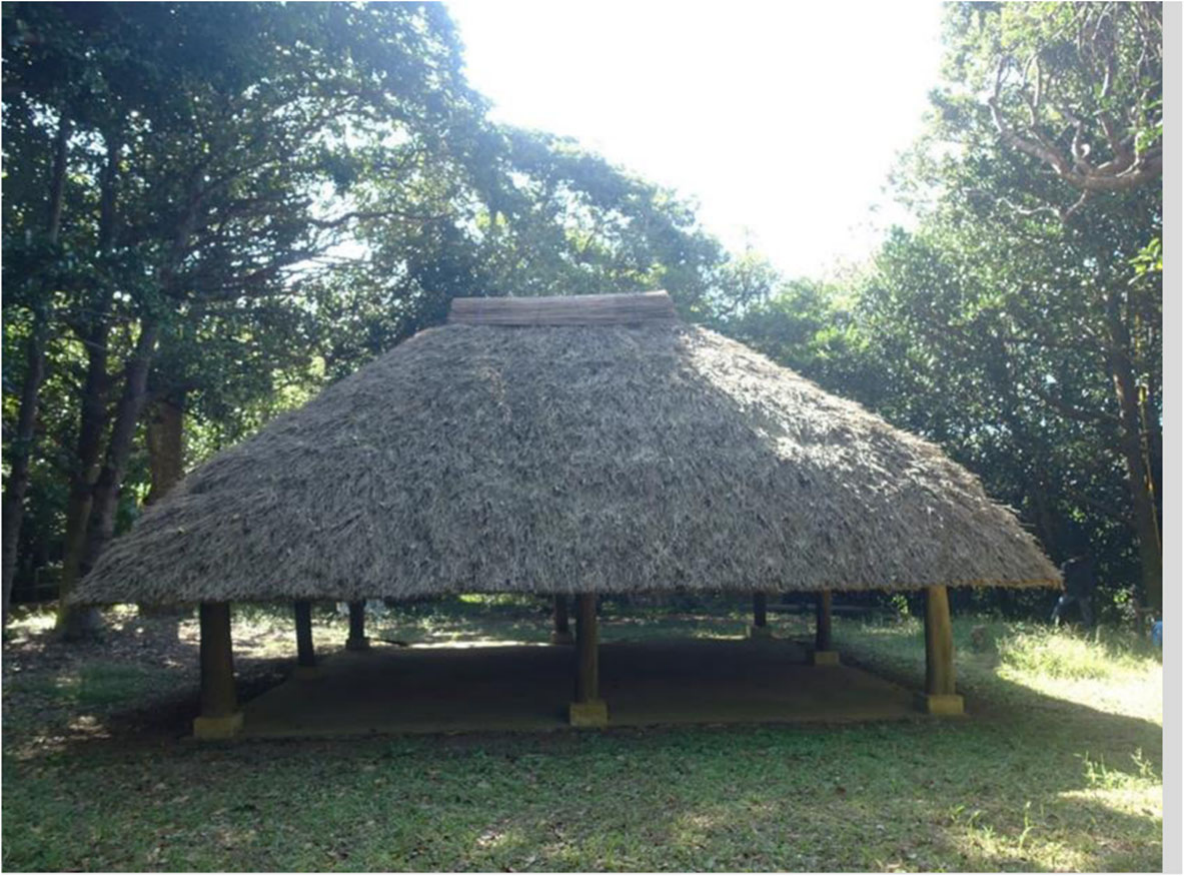
A traditional style sacred site of thatch-roofed *ashagi*, enclosed by an old-growth forest dominant by Fukugi trees (*Garcinia subelliptica*) and Akagi (*Bischofia javanica*) trees

Old Fukugi trees/forests found in sacred sites can be categorized into two types. One type contains only planted Fukugi trees, such as the Tōbaru *utaki* and *ashagi* (Fig. 5), the Nakadomari *ashagi* of Kunigami Gun, and in Furugen the *utaki* of Nakagami Gun (Table 3). The second type is a mixture with other native tree species such as *Bischofia javanica* and *Ficus microcarpa*, and includes Hiji *ashagi* (Fig. 6) and Kannonji (the Goddess of Mercy) Temple, Kim Town. Hiji *ashagi* is located inside the Kodamamori forest, east of the hamlet. Approximately 20 large Fukugi trees were found dispersed within the natural forest and have been well preserved since the origin of the hamlet. The average DBH of Fukugi trees in these four sites was estimated to be over 40 cm. Fukugi trees were the tallest in the Hiji *ashagi* since they were growing inside a thick natural forest. The other three sacred sites were located inside the hamlet surrounded by Fukugi trees. We observed that all Fukugi trees were growing in a circle. Thus, we assumed that the Fukugi trees were planted to enhance the sacred environment as internal circles in the natural forests.

### Heritage trees

In Japan, well-known large trees and old-growth trees were selected and archived from the perspective of “ancient trees living together with the local residents.” A heritage tree is typically large and old, with unique values that are closely interwoven with local life. The major criteria for heritage tree designations are age, rarity, and size as well as esthetic, botanical, ecological, and historical values of the trees [31]. In Okinawa, large trees/old-growth trees with high academic value, or cultural value derived from history and tradition, are designated as heritage trees at the municipal level.

**Fig. 7:**
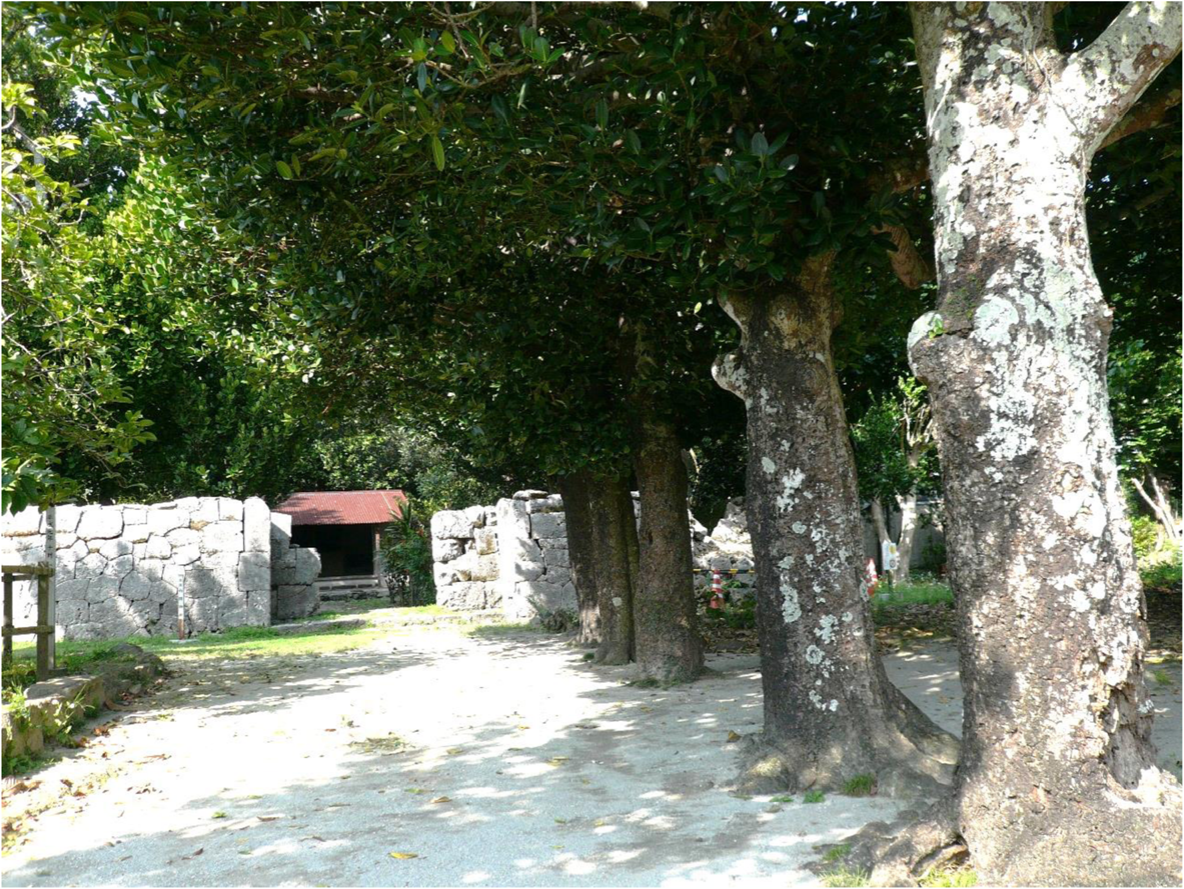
Fukugi trees in the cultural propriety

Thirteen sites of Fukugi trees/forests have been designated as heritage trees in Okinawa (Table 4) [32]. These heritage trees include large trees in historic sites (1), sacred places (6), and remnant homestead windbreaks (6). The largest Fukugi tree is located in the Uchima udun (Fig. 7) and was estimated to be 364 years old (surveyed on March 12, 2009). Udun is the ruin of a mansion inhabited by a relative of the Ryukyu King, a prince, or an Aji (a title and rank of nobility immediately below a prince). Uchima udun was designated as a national cultural property in 2011. It is believed that the Fukugi trees were planted in 1736, when the main building was changed from a bamboo windbreak to a Fukugi tree. Currently, 49 large trees remain at the site [33].

**Table 4:** Dimensions of Fukugi trees/forests designated as heritage trees in Okinawa Prefecture

Designated year	Place	Municipality	Properties	Dimensions of the biggest Fukugi tree		Survey date
				Diameter at breast height (cm)	Tree height (m)	
2003	Uchima Udun	Nishihara Town	Sacred site	81	14.1	Mar. 12, 2009
2004	Kannon Temple	Kim Town	Sacred site	90.5	12	Dec. 27, 2009
2004	Tonaki Bansho (village public office) ruins	Tonaki Village	Historic site	60.3	14	Jul. 2010
2005	Kijoka	Oogimi Village	Remnant homestead windbreak	41.4	6	Nov.2009
2005	Furugen Ugan	Yomitan Village	Scared site	87	13.6	Nov. 2009
2005	Nagahama Village	Yomitan Village	Remnant homestead windbreak	64.5	9.5	Nov. 2009
2005	Gushikami	Yaese Town	Remnant homestead windbreak	76.5	8.6	Nov. 2009
2006	Bise	Motobu Town	Remnant homestead windbreak	72.6		Aug. 2008
2006	Ungusuku Utaki	Tamara Village	Sacred site	68	9.3	Mar. 2010
2007	Uineetsuzu	Tamara Village	Sacred site	70	12	2007
2007	Minna utaki	Tamara Village	Sacred site	58.9	8.2	Mar. 2012
2012	Ige	Uruma City	Remnant homestead windbreak	69.4	12	2012
2013	Agarie	Ie Village	Remnant homestead windbreak	76.2	11.9	Dec. 12,2009

### Symbol trees

The cultural and esthetic values of this species have been highly appreciated, resulting in the species being selected as a city tree, town tree, or village tree in eight municipalities. Among the 41 municipalities (cities/towns/villages) in Okinawa Prefecture, eight municipalities have designated Fukugi trees as their symbol trees (Table 5) [34, 35]. Japan has three levels of governments: national, prefectural, and municipal. There are four types of local government municipalities in Japan that are based on population size: cities, towns, villages, and special wards (the *ku* of Tokyo). In terms of other symbol trees in Okinawa, the Ryukyu kokutan (*Diospyros ferrea*) municipality ranks among the top 10, followed by the banyan tree (*Ficus microcarpa*), which was designated within five municipalities. However, no municipality has officially recognized a Fukugi tree as a symbol tree outside Okinawa Prefecture in Japan.

**Table 5:** List of counties that have designated the Fukugi tree as the city/town/village tree in Okinawa Prefecture

County	City	Population^a^
Naha City		317951
Kunigami Gun	Motobu Town	13113
	Onna Village	10789
Nakagami Gun	Yomitan Village	39615
Shimajiri Gun	Aguni Village	715
	Tonaki Village	393
	Kumejima Town	7271
Miyako Gun	Tarama Village	1111

^a^Population on March 1st; data source: Okinawa Prefectural Office [34]

Data source: http://www.midorihana-okinawa.jp/?page_id=90

**Fig. 8:**
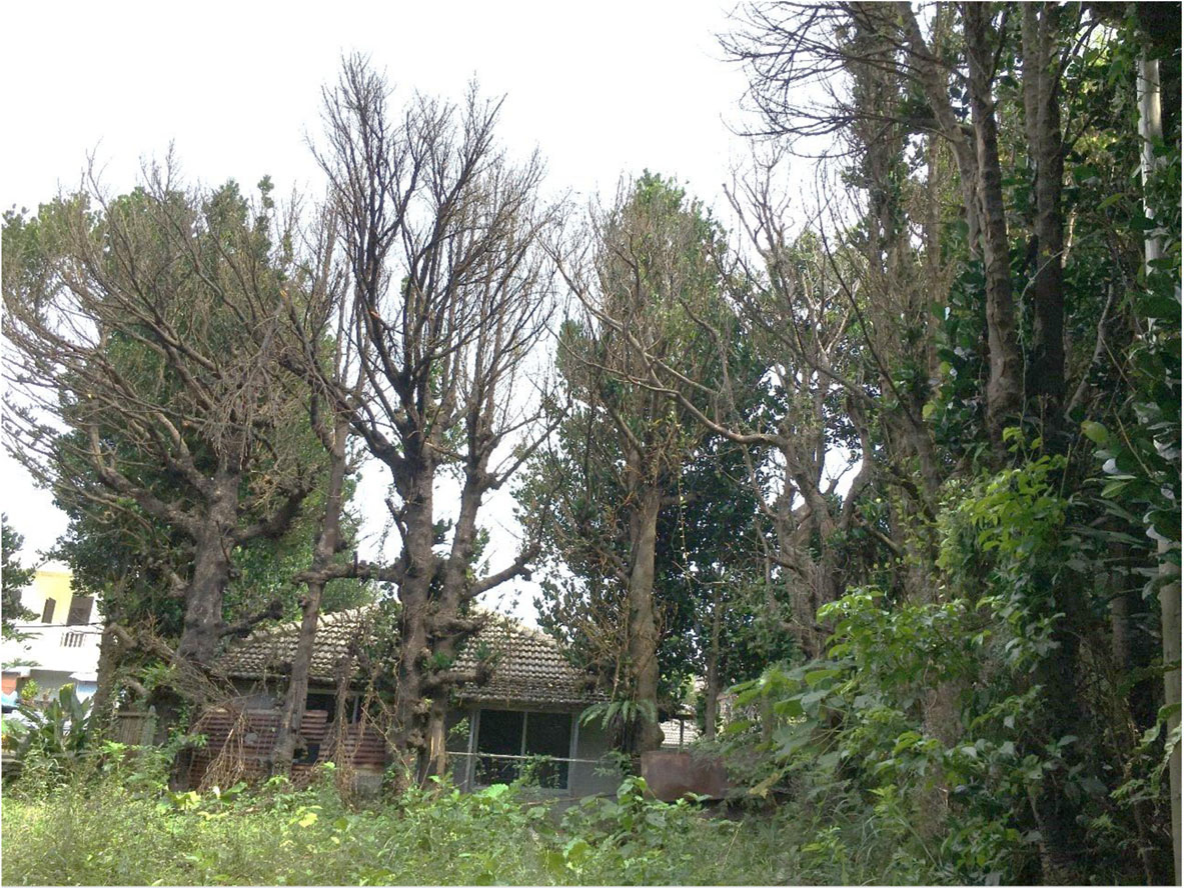
Fukugi trees prevented the fires caused by a short circuit in the front side from spreading to the neighbor’s house (photo taken in Imadomari, Nakijin Village, Okinawa Island, Sept. 11, 2020)

Tonaki, which is a small village on an isolated island in Okinawa, with a population of 355, an area of 3.75 km^2^, and a periphery of 12.5 km [36], was designated as a Fukugi tree village in 1992. The village homepage states that almost all houses have Fukugi trees as windbreaks (Fig. 2), with the trees being used to build and protect houses from salty sea water, strong winds, and fires (Fig. 8). Moreover, it is believed that Fukugi trees can bring happiness and prosperity and exist in harmony with the Okinawa subtropical landscape.

Island people highly value Fukugi trees as windbreaks and for ameliorating the microclimate. People in the smaller islands value trees more than people in mainland Okinawa [37]. Some residents even believe that cutting Fukugi trees will cast a curse and bring misfortune to a family [38]. A 50-year-old woman mentioned that she dared not break even a single branch of her neighbor’s Fukugi trees, which the old lady believed had a spirit dwelling in it.

## Discussion

The finding that Fukugi windbreaks provide significant ecosystem services and play a valuable role in biological conservation is consistent with other types of windbreaks, such as those found in agricultural lands [39], farmsteads [40], and in plains [41]. Windbreaks have also been used to reduce soil erosion [42], protect crops, reduce energy consumption, protect wildlife habitat [41, 43], mitigate greenhouse gas emissions [44], increase scenic beauty, supply firewood, and produce other types of timber products [45].

Our ethnobotanical research regarding Fukugi windbreaks revealed that a combination of the biophysical environment, as well as tradition and custom, play an essential role in the selection of tree species for windbreaks. Hence, we argue that Fukugi is a cultural keystone species in Okinawa. A cultural keystone species is defined as a species underpinning a culture and as one that plays an essential role in diet, in medicine, and/or in spiritual practices [46]. The geographical distribution of Fukugi windbreaks is almost the same as the geographical spread of Ryukyu Culture. Fukugi plays a unique role in people’s lives in the Ryukyu Archipelago. In addition to its important ecological functions, it is an important cultural and non-economic subtropical species. This study also implies that ethnographical approach is useful for assessing the diverse values of a plant species, as suggested by previous research pertinent to *Cinnamomum Camphor* in China [14].

It is assumed that the valuable function of Fukugi trees in protecting houses and farms has led to a strong feeling of solidarity and intimacy binding this species with island people, which distinguishes it from the other arbor species in the Ryukyu Archipelago. Consequently, people selected this species in the sacred sites as symbol trees for their locality. The presence of Fukugi landscapes has transformed the islands into pleasant and well-maintained land. It is widely accepted that natural environments, such as trees, are critical components of community pride and well-being [47]. Trees can be shared symbols that become part of the identity and features of a place [48].

Our findings also revealed the positive impacts of anthropogenic activities on the sustainability of woody species; the active utilization of tree species may have enabled the species to sustain. The Fukugi tree is a tropical evergreen species believed to be native to the Philippines, Taiwan, and Okinawa in Japan; however, they have been widely planted as windbreaks only in Okinawa [25]. We found that Fukugi trees may have disappeared in some Asian islands, such as the case in the Batanes island, if people believe that it is outdated, old-fashioned, or of not much use to humans. In the past, Fukugi was used as a windbreak, although Fukugi had also been used diversely as timber, a supplementary food resource, and as cloth dyeing material in the past. However, people do not utilize Fukugi trees anymore nowadays due to a high level of industrialization. The fading knowledge pertinent to diverse utilizations will jeopardize the sustainable uses of the species. A similar case was reported from the Utah state in the USA, in which Utahns cut down the heritage trees when they considered heritage trees as being old-fashioned, and sometimes ineffective [40]. Our findings support the conservation of biodiversity in terms of the interactions between humans and plants. Humans have a dominant influence on biodiversity and should focus their plant conservation efforts on the sustainable use, conservation, and sharing of the benefits derived from biodiversity [12, 49–51].

## Conclusions

*Garcinia subelliptica* (Fukugi) is an evergreen tropical tree that was first identified on Batanes Island, the Philippines, which has been extensively planted as homestead windbreaks and in coastal forests on the Ryukyu Archipelago, Japan. Impacts of climate change such as sea level rise, temperature rise, increase in extreme climate, and precipitation changes will affect the sustainable management of these old-growth trees [52]. Taking into consideration the potential increase of extreme climate, catastrophic disasters of typhoons, and even tsunamis, windbreaks will become increasingly important to guard people’s lives and properties in coastal areas.

However, strong feelings of solidarity and intimacy between Fukugi trees and humans have not been properly inherited by young generations. Fukugi trees have been attributed to tremendous spiritual or symbolic value by Ryukyuan culture in the past. Human culture is a crucial factor to the success of biological conservation and ecological restoration, to recognize and focus on cultural values of keynote species should be emphasized in conservation schemes [53].

Japan’s current framework of cultural properties can be applied to conserve homestead windbreaks in Okinawa. A national designation will help to raise local people and local governments’ awareness regarding protecting old-growth Fukugi trees and enable the national government to take various measures to protect cultural properties by providing subsidies for preservation, repairing, or public management of historic sites. Another means for conservation is to develop a more alternative utilization of the large number of Fukugi trees. Scientists have successfully extracted active chemical compounds from the leaves, fruits, seeds, wood, bark, and roots of Fukugi trees. Therefore, because humans are a dominant factor in the survival of this species, the utilization of bark, roots, flowers, and fruits should be part of any conservation strategy.

The present study is not free of limitations. More interviews are needed to clarify the historical, ecological, and cultural significance of *G. subelliptica* to the Ryukyu Island people. More islands and villages should be included, taking into consideration the wide distribution of Fukugi trees on the Ryukyu Archipelago. A thorough study of secondary data and field surveys from southern Taiwan is needed to explore the ecocultural values of tropical and subtropical Asia outside of Japan.

## Data Availability

The datasets used and/or analyzed in the current study are available from the corresponding author upon reasonable request.

## Appendix

Semi-structured interview participant list (gender, age at interview, and date of interview):

1. Bise Hamlet, Motobu Town, Kunigami District
  - K1., male, 68, 03 January 2015
  - M., male, 66, 19 February 2015
  - K2., male, 66, 28 February 2015
  - M2, male, 60s, 28 February 2015
2. Imadomari Hamlet, Nakijin Village, Kunigami District
  - U., male, 60s, 04 May 2015
  - S.M., male, 30s, 18 December 2020
  - S.T., female, 60s, 18 December 2020
  - T., mail, 40s, 19 December 2020
3. Hateruma Island, Toketomi Town, Yaeyama District
  - H., male, 60s, 26 December 2015
  - S, male, 60, 26 December 2015
  - K., male, 68, 27 December 2015
  - M., female, 82, 27 December 2015
4. Tarama Island, Tarama Village, Yaeyama District
  - T., male, 61, 27 April 2015
  - I., male, 60s, 28 April 2015
  - F., male, 70, 28 April 2015
5. Aguni Village, Shimajiri District
  - M., male, 50s, 18 April 2015
  - I., male, 62, 19 April 2015
  - T., male, 60s, 20 April 2015
  - S., male, 62, 20 April 2015
  - S., male, 34, 20 April 2015
6. Taketomi Island, Taketomi Town, Yaeyama District
  - A., male, 88, 29 September 2016
  - U., male, 60s, 30 September 2016
  - M.S., male, 90s, 28 December 2020
  - M.R., male, 90s, 28 December 2020
  - M.K., male, 50s, 28 December 2020
  - N., male, 80s, 28 December 2020
